# Micrometer Positioning Accuracy With a Planar Parallel Continuum Robot

**DOI:** 10.3389/frobt.2021.706070

**Published:** 2021-07-02

**Authors:** Benjamin Mauzé, Guillaume J. Laurent, Redwan Dahmouche, Cédric Clévy

**Affiliations:** FEMTO-ST Institute, University Bourgogne Franche-Comté, Besançon, France

**Keywords:** soft robots, continuum robots, cosserat-rod, kirchhoff-rod, parallel robots, micro-positioning, robot calibration, accuracy

## Abstract

Parallel Continuum Robots (PCR) have several advantages over classical articulated robots, notably a large workspace, miniaturization capabilities and safe human-robot interactions. However, their low accuracy is still a serious drawback. Indeed, several conditions have to be met for PCR to reach a high accuracy, namely: a repeatable mechanical structure, a correct kinematic model, and a proper estimation of the model’s parameters. In this article, we propose a methodology that allows reaching a micrometer accuracy with a PCR. This approach emphasizes the importance of using a repeatable continuum mechanism, identifying the most influential parameters of an accurate kinematic model of the robot and precisely measuring them. The experimental results show that the proposed approach allows to reach an accuracy of 3.3 µm in position and 0.5 mrad in orientation over a 10 mm long circular path. These results push the current limits of PCR accuracy and make them good potential candidates for high accuracy automatic positioning tasks.

## 1 Introduction

Parallel Continuum Robots (PCR) are a recent and rising type of robots introduced in (Bryson and Rucker, 2014). They are composed of flexible slender elements arranged in parallel and linked to a platform. Resulting robots present a higher rigidity than soft robots while keeping most of their advantages. This structure provides higher safety than common industrial robots thanks to the flexibility of the PCR’s limbs. For instance, Campa *et al.* proposed a planar PCR to perform safer collaborative robot applications (Campa et al., 2019). The flexibility of the limbs provides large continuous deformations that allow for a large workspace. Yang *et al.* proposed a continuum version of the Delta robot whose continuous joints allow larger rotations than usual joints (Yang et al., 2018). Flexible elements allow to reduce the number of joints and eliminate the mechanical plays (Black et al., 2018). Reducing the number of joints and mechanical parts also facilitates the miniaturization of the designed robot. For example, the continuum Steward-Gough platform introduced in (Bryson and Rucker, 2014) had its dimensions reduced in order to create a surgical PCR whose platform (a gripper) is around 10 mm diameter (Orekhov et al., 2017). This small prototype illustrates the interest and the capability of PCR to perform applications inside confined space (like the human body) where instrumentation and sensor-based controls are difficult to implement. To execute those applications, an accurate PCR could be a solution. However, the reachable accuracy of those structures is still an open question which needs more investigations. The objective of this paper is to propose a methodology to reach a high accuracy with PCR by measuring, identifying and understanding the influence of the different parameters, and have an estimate of a typical level of accuracy that a PCR can reach.

The accuracy corresponds to the difference between commanded poses and the barycenter of attained ones (ISO 9283, 1998) in position and orientation. A high accuracy enables to position the end-effector of the PCR thanks to its model and allows to successfully perform automated tasks. The accuracy of a robot depends on three key elements: its repeatability, the correctness of its model and the knowledge of the model’s parameters. The repeatability corresponds to the deviations of the reached poses for a same command, and mainly depends on robot’s design, the quality of its mechanical structure and its actuation system. A repeatable robot is essential to have a predictable behavior and thus to model it. The modeling of continuum robots appears especially challenging because of their virtually infinite number of Degrees-of-Freedom (DoF) and their highly nonlinear behavior due to the large deformations. Two modeling approaches of parallel continuum robotics are widespread in the literature: physical-based models and black-box models such as neural networks. Using an artificial neural network, Wu *et al.* reached a positioning accuracy of 5 mm. One of the drawbacks in using black-box models is that they could not identify why the positioning accuracy was low (Wu et al., 2017). This is one of the reasons why most of the studies use physical-based models.

We can distinguish two categories of physical-based models: high fidelity distributed parameter models and low fidelity parameter models (Rone and Ben-Tzvi, 2014). The low fidelity parameter models use strong hypothesis to reduce the number of parameters and to facilitate the modeling and the identification of its parameters. For instance, constant curvature models represent continuum deformations by considering that flexible element shapes are circle arcs (Nuelle et al., 2020; Lilge et al., 2021). Those approaches are interesting for their simplicity but seem not suitable for high positioning accuracy. For this reason, physical-based models with high fidelity distributed parameters and variable curvature assumption are preferred. Depending on the structure, Cosserat-rod-based models (Trivedi et al., 2008; Orekhov et al., 2017; Till and Rucker, 2017; Black et al., 2018) or Kirchhoff-rod-based ones (Takano et al., 2017; Altuzarra et al., 2019; Altuzarra and Merlet, 2019; Campa et al., 2019) gained consensus because of their ability to predict the shape and forces of the rods. In the case of tendon-actuated continuum structures, Rao *et al.* proposed guidelines to choose a model depending on the targeted application (Rao et al., 2021). Such guidelines do not currently exist for parallel continuum structures.

In addition to the modeling, the measurement and the identification of the model’s parameters are also important. Indeed, to get an accurate prediction of the robot’s behavior, the model’s parameters need to be close to their real values. To address this issue, robot calibration is usually performed. However, as Wu *et al.* pointed out, the calibration of PCR is more complex than for regular parallel robots (Wu and Shi, 2019). One example is the absence of an analytical model. This complexity generally conducts to choose a simpler model to facilitate the identification step (Dehghani and Moosavian, 2013) or to measure the parameters rather than identifying them (Orekhov et al., 2016). The chosen model, the measurement and the identification of its parameters have a strong and deeply intertwined impact on the robot’s accuracy.

Recent studies quantified the accuracy of several PCR that are gathered in Table 1. Even if it is difficult to compare them because of their different designs, those values give an overview of the expected reachable accuracy of current PCR using different models. To be more representative to the PCR’s design, the positioning accuracy is generally expressed in percent of the nominal or the mean dimension of the continuum flexible segment. For example, Orekhov *et al.* obtained a mean positioning accuracy of 2.8% (1.19 mm) and a mean orientation accuracy of 3.81° after identifying extrinsic parameters of a 6-Degrees-of-Freedom (DoF) robot (Orekhov et al., 2016). The accuracy is interesting for a spatial PCR even if it is limited by the actuation system whose positioning accuracy is about 0.1 mm. The actuation system is also one of the main uncertainty sources for the positioning accuracy calculated from the data presented in (Yang et al., 2018). In the studies of Wu *et al.* (Wu and Shi, 2019; Wu et al., 2017), several reasons were pointed out like the small construction and assembly tolerances or the friction forces that were not took into consideration. Nuelle *et al.* proposed a study of a tendon-actuated planar PCR and reached an accuracy of 1.4% (1.8 mm) after identifying all model’s parameters thanks to a calibration process (Nuelle et al., 2020). The shown accuracy was limited by the constant curvature approach and by the actuation and the robot design, which suffer from gear backlash and static-friction.

**TABLE 1:** Mean orientation $AP_{\theta }$ and positioning accuracy $AP_{P}$ (in percent of the nominal or mean dimension of the continuum flexible segment) for different PCR designs.

Design	Image	DoF	Structure	Models	Calibration	$AP_{P}$ (%)	$AP_{\theta }$ (°)
Orekhov et al. (2016)	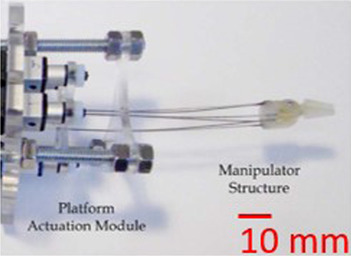	6	6 PF	Cosserat	No	2.8	3.81
Yang et al. (2018)	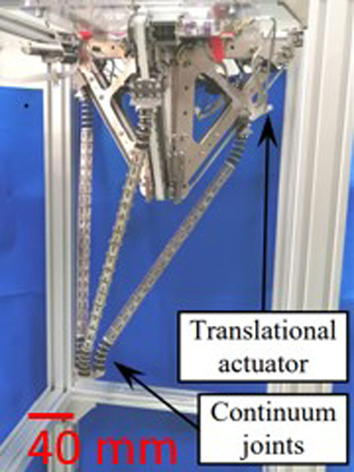	3	3PFF	Cosserat	No	0.1	-
Wu et al. (2017)	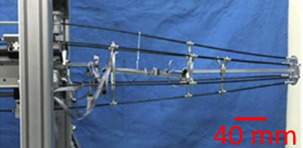	3	Multi	Neural Network	No	0.43	-
Wu and Shi (2019)			Constraint	Cosserat	Yes	0.8	-
Nuelle et al. (2020)	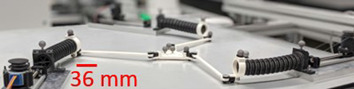	3	3PFR	Constant Curvature	Yes	1.4	1.1

**FIGURE 1:**
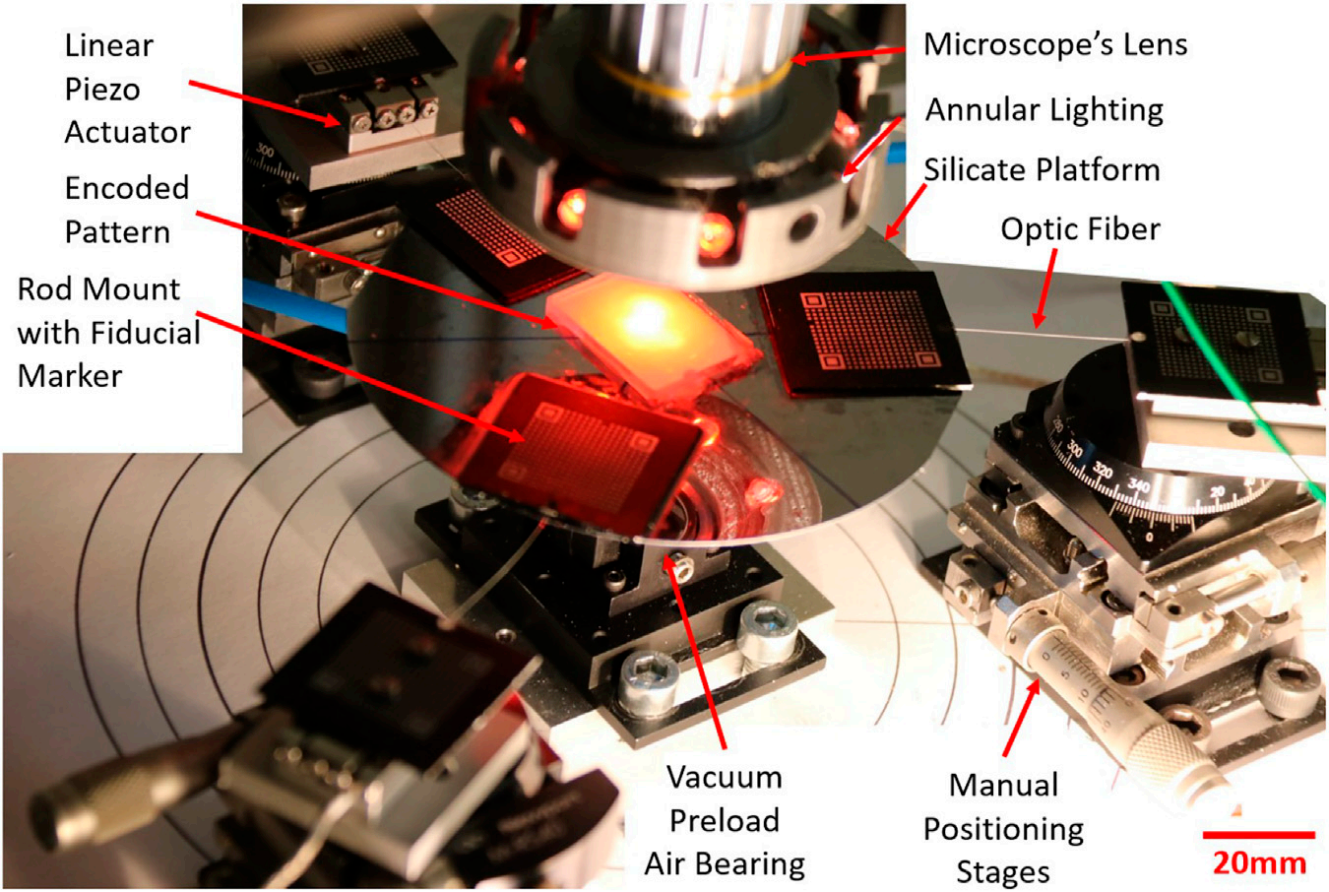
Picture of the XYΘ parallel continuum robot.

To address the challenge of getting accurate PCR, the proposed approach consists in five key elements. The first one consists in using a repeatable PCR. The robot recently introduced in (Mauzé et al., 2020) was able to provide an outstanding repeatability of 9.13 nm in position and 1.2 µrad in orientation. Choosing a planar architecture allows using a very resolute and long range multidimensional measurement system which facilitates the understating of the proposed methodology. Thanks to its mechanical structure, this robot seems suitable to study the accuracy reachable by PCR. This three DoF XYΘ planar parallel continuum is illustrated in Figure 1. The second point is the use of a correct mechanical model. The measurement of the model’s parameters is facilitated by the PCR design which also anticipates the calibration process. That is why fiducial markers, third element of the proposed methodology, are introduced. Those fiducial markers, placed at specific locations of the PCR, enable to efficiently measure the nominal values and to estimate the uncertainties of the different models’ parameters. The fourth element is a sensitivity analysis which coupled with the uncertainties allows to determine the most influential parameters. The last key point is the calibration process of the robot.

The next section presents the model of the PCR and the sensitivity analysis. This analysis enables to understand the role of each parameter and to identify the most influential ones. Those parameters require a special attention during the design and the calibration processes of the robot. The robot prototype and the experimental setup are described in *Section 3*. The models’ parameters measurement step with an uncertainty analysis and the calibration process are respectively presented in *Section 4 and 5*. The last section demonstrates the capability of the robot to perform desired trajectories and quantifies the accuracy using the identified parameters.

## 2 3-DOF Planar PCR Model

To study Parallel Continuum Robots’ (PCR) accuracy, we considered a high-grade repeatable robot illustrated in Figure 1. This robot is composed of three planar kinematic chains that are linked together to a rigid moving platform. Contrary to classical 3-PRR mechanisms which inspired this design, each chain is composed of a prismatic actuator and a flexible rod that deforms continuously. The three flexible rods transmit the actuators’ forces to the platform inducing its motions. Using the traditional naming convention, the flexible rod is denoted using the letter “F” and the resulting PCR design is then a 3-PF robot.

**FIGURE 2:**
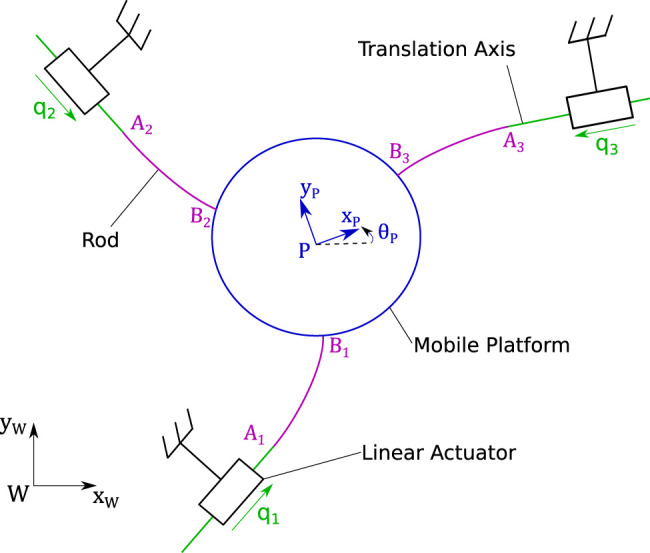
Kinematic diagram of the 3-PF planar Parallel Continuum Robot.

Figure 2 illustrates the kinematic diagram of the 3-PF robot. The actuation stages, through the prismatic joint values $q_{1}$, $q_{2}$, $q_{3}$, push and pull the rods to move the mobile platform. The rods are slender beams capable of continuous and large deformations. The movement of this mobile platform is restricted to planar displacements (*x*, *y* translations and θ rotation).

A global work frame $\left(W,x_{W},y_{W},z_{W}\right)$ is defined with the $z_{W}$ axis perpendicular to the robot’s base. A frame $\left(P,x_{P},y_{P},z_{P}\right)$ is attached to the mobile platform. The three rods are clamped to this platform at their distal ends $\left(B_{i},x_{B_{i}},y_{B_{i}},z_{B_{i}}\right)$. The proximal ends of the rods clamped to the actuator are defined by the frames $\left(A_{i},x_{A_{i}},y_{A_{i}},z_{A_{i}}\right)$.

In the following, we describe the model used to simulate the quasi-static behavior of the robot. First, the model of a single rod is detailed. Then, the three models of rods are linked together thanks to the static equilibrium of the platform. All those elements allow to implement the forward and the inverse kinematic models of the robot.

### 2.1 Rod Model

The PCR’s model depends mainly on the modeling of the rods and its correctness, that is why a Kirchhoff-rod-based model is used. This model supposed that shear and extension can be neglected. It is possible as the used slender elements have their cross-section more than a hundred times smaller than their lengths. The proposed structure remains in a plane so, only the planar case of this model is considered without losing the generality of the proposed approach which can also be adapted for a spatial robot.

**FIGURE 3:**
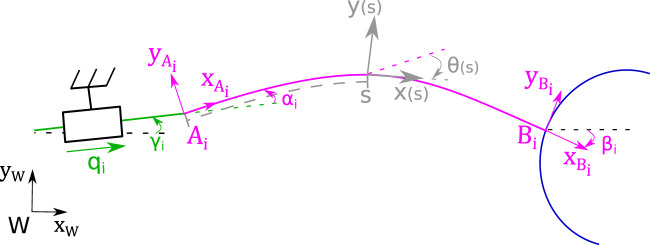
Detailed scheme of the *i*-*th* limb with frames and parameters.

The curvilinear abscissa is represented by the scalar parameter s∈[0,l] where *l* is the stress-free length of a rod. Along its arc length, $p\left(s\right)={\left[\begin{array}{c} x\left(s\right),y\left(s\right) \end{array}\right]}^{T}$ and θ(s) respectively define the cross-section centroid position and orientation in the frame attached to the proximal end of the rod. Figure 3 shows the entire model of a limb.

All involved differential equations can be gathered into the following system:

$$\left\{\begin{array}{c} \frac{dx\left(s\right)}{ds}\\ \frac{dy\left(s\right)}{ds}\\ \frac{d\theta \left(s\right)}{ds}\\ \frac{dn_{x}\left(s\right)}{ds}\\ \frac{dn_{y}\left(s\right)}{ds}\\ \frac{dm\left(s\right)}{ds} \end{array}\right\}=\left\{\begin{array}{c} \cos\left(\theta \left(s\right)\right)\\ \sin\left(\theta \left(s\right)\right)\\ \frac{m\left(s\right)}{EI}\\ 0\\ 0\\ n_{x}\left(s\right)\sin\left(\theta \left(s\right)\right)-n_{y}\left(s\right)\cos\left(\theta \left(s\right)\right) \end{array}\right\}$$ (1)

Where $n_{x}\left(s\right),n_{y}\left(s\right)$ are respectively the X and Y components of the internal force n(s),m(s) m(s) is the internal moment, *E* is the Young modulus of the rod material, and *I* is the second area moment of the rod cross-section which depends on the rod’s diameter *d*.

### 2.2 Forward and Inverse Kinematic Models

To model the quasi-static behavior of the PCR, a forward and inverse kinematic models are created. Both result from a numerical resolution of the previous equations considering the rod’s boundary conditions and the static equilibrium of the platform.

Those boundary conditions describe how the rods link the actuators to the mobile platform. They yield:

TPW=TAiW⋅TBiAi⋅TPBi (2)

where TAiW depends on the joints coordinates $q_{i}$, TBiAi corresponds to the transformation resulting from the integration of the rod’s equations TPBi is constant reflecting the rigid-body conditions between the distal ends of the rods and the mobile platform, and TPW depends on the desired position such as:

TPW=[cosθ−sinθxsinθcosθy001] (3)

To know the pose of the platform, the static equilibrium conditions are considered:

$$\left\{ \begin{array}{l} \overset{3}{\underset{i=1}{\sum }}\left[n_{i}\left(l_{i}\right)\right]-f_{P}=0\\ \overset{3}{\underset{i=1}{\sum }}\left[p_{B_{i}}\times n_{i}\left(l_{i}\right)+m_{i}\right]-p_{P}\times f_{P}-m_{P}=0 \end{array}\right.$$ (4)

where $f_{P}$, $m_{P}$ are the external force and moment applied on the platform in the work frame, where $n_{i}$, $m_{i}$ are the rods’ forces and moments applied on the platform in the work frame, $l_{i}$ is the length of the rod *i* and $p_{B_{i}}$ and $p_{P}$ are the positions of $B_{i}$ and *p* in the work frame.

Due to the coupling between the rods, there is no analytical solution of those equations considering the previous boundary and equilibrium conditions, thus, a numeral resolution is performed using a shooting method. This shooting method is based on a optimization problem where Eqs 2, 4 constitute its residual vector (Till and Rucker, 2017; Mauzé et al., 2020).

### 2.3 Sensitivity Analysis of the Models’ Parameters

The PCR accuracy depends on the values of the model’s parameters. To estimate the influence of the different parameters, a sensitivity analysis is performed. It also allows for identifying which parameters required more consideration. There are three kinds of parameters: the intrinsic rod parameters (the Young modulus *E* of its material, its diameter *d* and its length *l*), the ones representing the poses of the rods’ proximal and distal ends (respectively $A_{x_{i}},A_{y_{i}},\alpha_{i}$ and $B_{x_{i}},B_{y_{i}},\beta_{i}$), and the parameters $\gamma_{i}$ which represent the misalignments between the directions of translations and the rods’ orientation at the proximal ends.

The principle of the proposed sensitivity analysis is the following. An arbitrary set of 26 joints configurations is chosen in the center of the workspace. For each configuration, partial derivatives of the platform position considering the different parameters are numerically computed using a finite difference method. The maximal values of the partial derivatives among the configurations and the different rods are gathered in Table 2. All parameters are expressed in the SI base unit.

**TABLE 2:** Influences of the different models’ parameters (expressed in SI base unit) computed as the maximal finite difference for the different configurations and rods.

Parameters	$max_{i}\left(\frac{\partial x}{\partial param}\right)$	$max_{i}\left(\frac{\partial y}{\partial param}\right)$	$max_{i}\left(\frac{\partial \theta }{\partial param}\right)$
E	0	0	0
D	0	0	0
$d_{i}$	0.2	0.2	0.05$\times 10^{3}$
*α*_*i*_	1.3$\times 10^{-3}$	1.3$\times 10^{-3}$	0.4
*β*_*i*_	0.3$\times 10^{-3}$	0.4$\times 10^{-3}$	0.2
*γ*_*i*_	0.3$\times 10^{-3}$	0.3$\times 10^{-3}$	0.1
$l_{i}$	1.5	1.3	0.4$\times 10^{3}$
$A_{x_{i}}$	1.3	1.2	0.4$\times 10^{3}$
$A_{y_{i}}$	1.0	0.8	0.4$\times 10^{3}$
$B_{x_{i}}$	1.3	1.2	0.4$\times 10^{3}$
$B_{y_{i}}$	1.0	0.8	0.4$\times 10^{3}$

From the table results, three groups of parameters can be defined considering their influence on the model. The first group is composed of the Young modulus and the diameters of the rod. The Young modulus has no influence on the pose of the platform. It is the same observation for the diameter if the three parameters are equal. This assumption can be considered as true if the different rods are created from the same element. If one diameter is different than the other, it introduces an asymmetry and thus a small variation of the modeling. This difference of behavior is only visible for the diameters. Indeed, if all lengths (for example) are changed in the same time, the consequences on the model will be more important than if only one length was changed.

The second group is composed of the angular parameters $\alpha_{i}$, $\beta_{i}$ and $\gamma_{i}$. The influence of the parameter $\alpha_{i}$ is more important than the one of the other angular parameters. It can be explained by its role in the transmission of the actuator’s movement. To understand the influence in terms of orientation, considering a 1 mrad uncertainty on a $\alpha_{i}$ and the value of the table, the resulting positioning error for the model is about 1.3 µm. If this value is compared with a case-study of an uncertainty of 1 mrad made on the orientation of a rigid bar of 30 mm, the estimation of the position is about 30 μm at the bar’s end. So, it can be said that the influences of those angular parameters are relatively small. The elasticity, the deformation of the rod and the parallel structure reduced their influence.

The last group is composed of the parameters that are the most influential: the length of the rods $l_{i}$ and their end positions $A_{x_{i}},A_{y_{i}},B_{x_{i}},B_{y_{i}}$. The differences between the direction X and Y are essentially due to the asymmetry of the robot’s structure which is induced by the initial orientation of the rods.

This sensitivity analysis give information about the influence of the different parameters but also indicate the threshold of uncertainty where their influence can not be neglected anymore. To give an example of comparison between the parameters’ influence, considering the values of the table, an uncertainty of 1 mrad observed on a proximal end’s orientation $\alpha_{i}$ has the same influence of an uncertainty of 1 µm observed on the estimation of the X coordinate of the proximal end position $A_{x_{i}}$.

In order to get an accurate robot, the parameters whose influences are the most important on the model need to be estimated as precisely as possible. The next section will present the PCR design which takes into account the presented results by introducing fiducial markers. The objective of those markers is to reduce the measurement uncertainty of the most influential parameters.

## 3 Robot Design and Measurement System

To validate the modeling of the robot, a prototype has been built by taking into account the previous sensitivity analysis. To perform this comparison between the prototype’s experimental behavior and its model, the PCR poses and its models’ parameters have been measured by two complementary vision measurement systems. This section introduces the robot design and the associated measurement system.

### 3.1 Robot Design

The robot is composed of a mobile platform coupled to three actuated continuum limbs.

Each limb is assembled on a stack of three manual precision stages, two translation stages (Newport SDS-40) and a rotation stage (Newport M-RS40). These manual stages are used to adjust the position and the orientation of each actuator fixed above them.

The actuators are SmarAct (SLC-1730-S-HV) positioning stages. Their repeatability is below 30 nm and their range is 21 mm. Those actuators possess accurate optical sensors and a repeatable behavior that are necessary to get accurate inputs for the forward kinematic modeling of the PCR.

The rods are optical fibers stripped of their plastic part. Those fibers are in fused silica whose Young Modulus *E* is about 69 GPa. They are able to do large deformations without viscous effect thanks to their elasticity. They are 125 µm in diameter and about 30 mm in length. Those rods are connected to the actuators and to the platform by the mean of rod mounts.

The rod mounts are the key elements to define precisely the position of the rods’ ends and thus their length. Each rod mount is drilled on one side to clamp the rods. On the top face of the mount, a fiducial marker (QR-Code-like pattern) is engraved. Thanks to its manufacturing, the transformation between the center of the fiducial marker and the hole where the rod are inserted is known with a small uncertainty. Thus, knowing the pose of the fiducial marker will enable to know the relative pose of the proximal and the distal ends of the three rods (more details will be given in the next section). The proximal rod mounts are fixed on the actuators and the distal rod mounts are glued on the mobile platform.

The platform is a 100 mm silicon wafer lifted by a 50 mm diameter air bearing (S205001) from the IBS company. This air bearing avoids friction and is preloaded to maintain a stable elevation of the platform (± 5 µm). A manual linear stage (Newport M-DS25-Z) allows to adjust the level of the air bearing and to get the required planarity of the entire PCR.

### 3.2 Vision Setups

**FIGURE 4:**
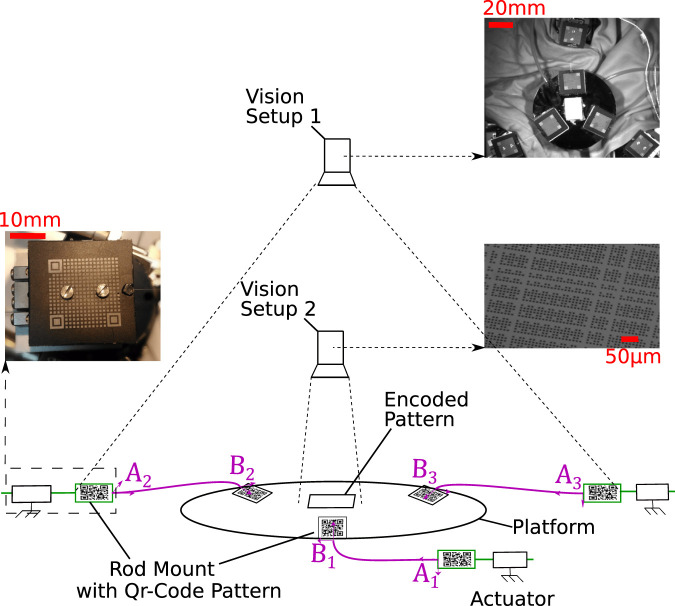
Scheme of the experimental setup with the several measurement vision systems.

The knowledge of the initial configuration of the robot is mandatory for an accurate simulation and position control of the robot. For this purpose, two vision measurement systems have been set up as shown in Figure 4.

The first one relies on the localization of fiducial markers embedded on the rod mounts. The position of these fiducial markers are tracked over a large field with a camera (IDS UI3008CP-3) mounted with a 50 mm lens. This vision setup is used to get accurate measurements of the different models’ parameters of the robot. More details will be given in the next section.

The second vision measurement system is dedicated to the measurement of the Cartesian pose of the platform with a very high resolution. This system is composed of a camera with a microscope tube, a x20 lens from Mitutoyo and a pseudo-periodic pattern glued on the mobile platform of the PCR. This system is able to measure the *x*, *y* position and the θ angle of the platform with a sub-nanometer resolution (Andre et al., 2020). Both cameras are supported by a robust gantry in order to minimize mechanical noise.

## 4 Parameters Measurement

After the PCR’s model is created, the PCR accuracy will depend on the value of the model’s parameters. The closer the value of the parameters will be, the higher will be the PCR’s accuracy. The measurement of those parameters is an essential step. Specially those whose influence, resulting from the sensitivity analysis, is important. This section explains how nominal values and measurement uncertainties are obtained in the aim to have an accurate control of the robot.

The different parameters are related to the rods, the position of the rods’ ends and the actuators’ direction of displacement. The quantification of the uncertainty will define intervals which will give more information about the potential modeling errors, and help to identify the parameters during the calibration process.

### 4.1 Rod Parameters

The diameter of the rods is measured thanks to a caliper which has a measurement uncertainty of 20 µm. The length of the rods is measured from images taken with the first vision setup. The measurement uncertainty is estimated to three pixels. Considering the pixel/meter ratio, this uncertainty is 126 µm. Considering the uncertainties values and the results of the sensitivity analysis, the rod diameters influence can be neglected while the rod lengths need to be identified carefully.

### 4.2 Rod End Positions

The sensitivity analysis points out that the rod end positions are influential parameters. Those positions depend on the rod mounts position and the clamping conditions. All rod mounts positions can be directly measured with the first vision setup and a specific fiducial marker detection process. This algorithm gives the Cartesian coordinates of all fiducial markers in the camera frame. The six rod mounts can be differentiated thanks to missing squares in each fiducial markers (coding principle). The resolution of the measurement is less than 0.25 µm for X and Y translations and 0.5 mrad for rotation.

As the transformation between the center of the fiducial marker and the clamping hole is known, the Cartesian position of the proximal end *A* and the distal end *B* of each rod can be deduced from these markers poses. By construction, the transformations between the center of the fiducial marker and the clamping point of the rod are defined by two translations of 14 mm and 250 µm in the *X* and *Y* directions. The machining accuracy is about 6% of this transformation leading to uncertainties of respectively 840 and 15 µm. By acquiring an image close to the rod mount and the clamping area and considering the resulting pixel/meter ratio, the uncertainty in the *X* direction is reduced to 160 µm. Due to the value of the uncertainties and the influence of those parameters, they will need a special cares during the PCR calibration process.

### 4.3 Actuator’s Direction of Displacement

**FIGURE 5:**
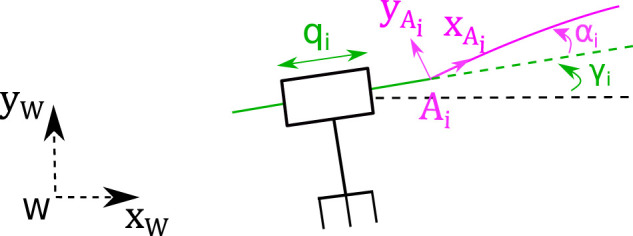
Detailed scheme of the misalignment between the rod mount and the translation axis of the piezo stage.

The last parameters that need to be measured are assembling defaults between the actuators and the rod mounts (Figure 5). The angle of the stage axis in the work frame, γ_*i*_, is measured using 1 mm displacement of the stage with a step size of 125 µm. Each pose of the pattern is recorded during this displacement. The angle of the regression line through these points gives a precise measurement of γ_*i*_. Then, the difference between this angle γ_*i*_ and the pattern pose provides the misalignment angle α_*i*_. The uncertainty for both angles is estimated to 5 mrad. Considering their small uncertainties, the parameters $\beta_{i}$, $\gamma_{i}$ will be neglected and only the parameters $\alpha_{i}$ will be considered.

Thanks to this measurement process, nominal values of the different parameters and their uncertainties are known. This knowledge is useful to prepare the calibration of the PCR.

## 5 Robot Calibration

**FIGURE 6:**
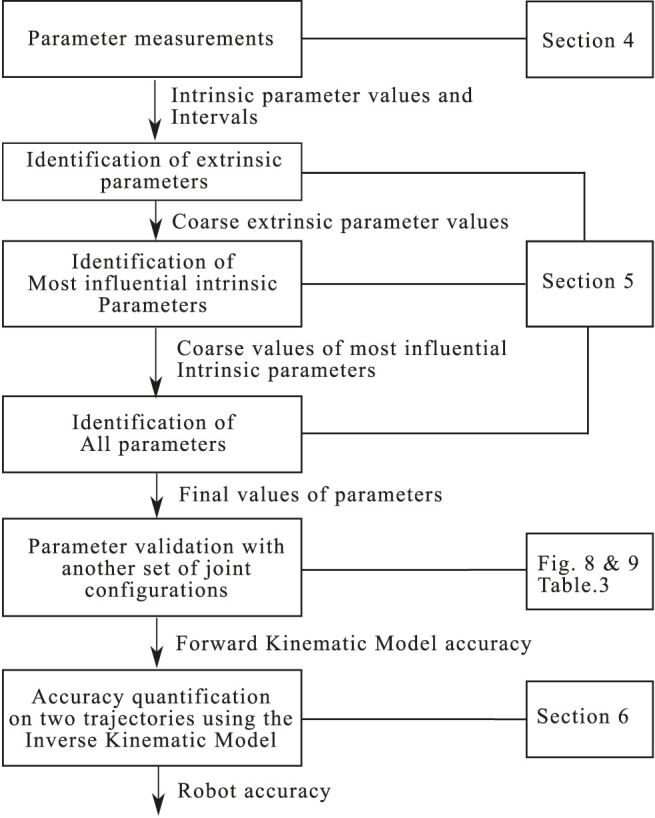
Flowchart of the parameter identification strategy.

**FIGURE 7:**
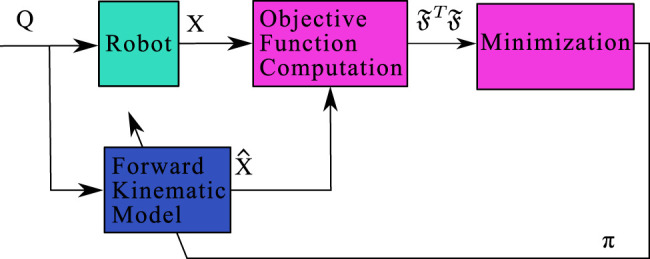
Block diagram of the identification principle using the Forward Kinematic Model. Considering a set of joints configuration **Q**, the parameters π of the model are changed such as the residual of the objective function Π is minimized.

Even with an accurate measurement process, the addition of small uncertainties reduces the PCR’s accuracy. To reduce those errors and obtain a better fitting between the experimental behavior and the model a calibration process has been implemented. In this article, three identification steps are used in this calibration process described in Figure 6. The principle of one identification is illustrated in Figure 7. The new values of the parameters are usually obtained thanks to a minimization of an objective function. This section details the different considered parameters, the definition of this objective function, the identification strategy, the experimental results and the obtained position and orientation errors.

### 5.1 Extrinsic and Intrinsic Parameters

There are two kinds of parameters considered for the identification step of the calibration process: the intrinsic parameters $\pi_{r}$ (defined in Table 2) which correspond to the model’s parameters, and the external parameters which depend on the measurement system (whose uncertainties are at the nanometer level) used to get the pose of the PCR.

The experimental poses are acquired thanks to the second vision setup as shown in Figure 4. They correspond to the 2D poses of a frame attached to the pseudo-periodic pattern with regards to the camera frame TMSe. These measured poses are used to get the experimental Cartesian pose of the platform in the work frame TPW as follows:

TPW=TSeW⋅TMSe⋅TPM (5)

The transformations TSeW and TPM respectively correspond to the transformation between the work frame and the camera frame, and to the transformation between the pseudo-periodic pattern frame and the platform frame. TSeW and TPM are constant but cannot be measured directly and must be identified. The six corresponding extrinsic parameters are gathered in the vector $\pi_{ext}$. The experimental pose TPW defined by $X={\left[x,y,\theta \right]}^{T}$ has to be compared with those resulting from the simulation defined by $\hat{X}={\left[\hat{x},\hat{y},\hat{\theta }\right]}^{T}$.

### 5.2 Objective Function

The parameters identification is based on a minimization of an objective function using a least squares algorithm. This algorithm is defined by the sum of the squares of the differences between the experimental Cartesian poses of the robot and the simulated ones using the forward kinematic model. Both experimental and simulation poses depend on a set of *n* joint coordinates $Q=\left[q^{1}\ldots q^{n}\right]$ where $q^{j}={\left[q_{1}^{j},q_{2}^{j},q_{3}^{j}\right]}^{T}$ is the *j*-th joint vector in the set. Considering the measured and simulated poses which depend on $q^{j}$ and $\pi ={\left[\pi_{r},\pi_{ext}\right]}^{T}$, the vector of residuals is defined by $ℑ={\left[{ℑ}^{1}\ldots {ℑ}^{n}\right]}^{T}$ where ${ℑ}^{j}$ is:

$${ℑ}^{j}\left(\pi ,q^{j}\right)=\left[\begin{array}{ccc} \alpha *\left({\hat{\theta }}^{j}-\theta^{j}\right) & \left({\hat{x}}^{j}-x^{j}\right) & \left({\hat{y}}^{j}-y^{j}\right) \end{array}\right]$$ (6)

where α is a weighting factor. This factor has been chosen such as the residuals in orientation and position were proportional to the repeatability of the robot.

Using this function, the identification problem can be formalized as:

$$\begin{array}{c} \underset{\pi }{\min imize}\;{ℑ}^{T}ℑ\\ subject\;to\;\pi \in \left[\begin{array}{c} l_{b},u_{b} \end{array}\right] \end{array}$$ (7)

where, $l_{b}$ and $u_{b}$ are the lower and upper bound values of the parameters, provided by the sensitivity analysis.

The minimization of this optimization problem is performed using the built-in-function lsqnonlin from Matlab software.

### 5.3 Calibration Process

To increase the PCR’s accuracy, the parameters can be identified after their measurements. This initial measurement of the parameters enables the reduction of the parameters’ uncertainty intervals. Small intervals help to avoid some local optima and ensure consistent parameters during the minimization. Moreover, the sensitivity analysis helps to distinguish the most influential parameters that should be identified in priority. Indeed, the parameters could not be identified at the same time if their values are too far from their true values. For this reason, the identification is carried out in three steps using the same experimental data.

The parameters that need to be identified first are the extrinsic parameters $\pi_{ext}$. Indeed, those parameters cannot be measured and have only been coarsely estimated. Then, after setting the extrinsic parameters, the most influential robot’s parameters can be identified in a second step. From the analysis, those parameters are the length of the rods, the orientation of the translation direction of the actuator and the position of their ends. The two first steps aim at reducing substantially the errors but the values of the parameters may not be optimal yet, because some parameters could compensate for the uncertainties of the others. In the last step, all parameters are re-identified together, after the identification of the most influential parameters. This final optimization begins with an initial set of parameters that assumed to be relatively closed to their true values.

### 5.4 Calibration Results

**FIGURE 8:**
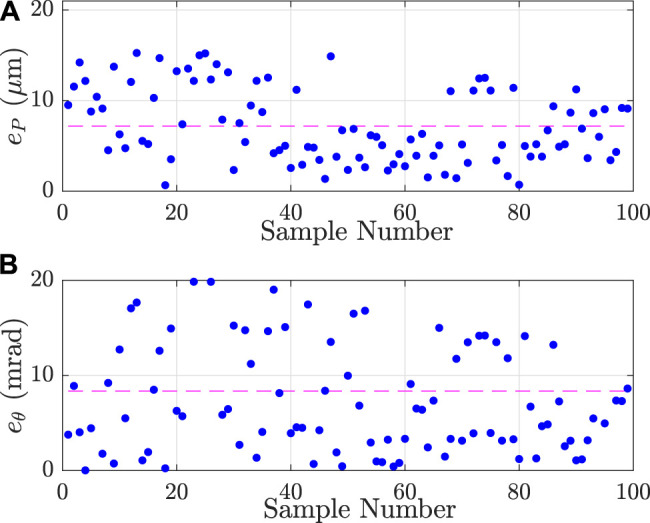
Positioning **(A)** and orientation **(B)** errors of the model (respectively $e_{P}$ and $e_{\theta }$) for the different samples of the calibration set of joints coordinates. The dashed-lines are the mean values of positioning and orientation errors and respectively correspond to 7.19 µm and 1.7 mrad.

As previously explained, the joint coordinates have been chosen inside the robot’s workspace in which it has a nanometer repeatability. These joint coordinates are sent to the robot and the Cartesian coordinates of the platform are recorded. To ensure a good distribution in the available workspace, the joint coordinates have been randomly chosen using a 3-dimensional Poisson-disc sampling. A set of 99 joints coordinates, more than three times the number of considered parameters, is created to perform their identification. 56 joints coordinates are selected to validate the identified parameters. Figure 8 presents the result of the calibration process. With the warm-up cycle of the camera and the actuators, the experimental measurement of the calibration process lasts around 6 h. It shows the errors between the platform positions and orientations simulated with the nominal model (model before calibration) and the model after calibration using the experimental data.

The position error of the pose *j* is defined as the root mean square of the difference between the simulated position $\left({\hat{x}}^{j},{\hat{y}}^{j}\right)$ and the experimental one $\left(x^{j},y^{j}\right)$ such as:

$$e_{P}^{j}=\sqrt{{\left({\hat{x}}^{j}-x^{j}\right)}^{2}+{\left({\hat{y}}^{j}-y^{j}\right)}^{2}}$$ (8)

Similarly, the orientation error is:

$$e_{\theta }^{j}=\sqrt{{\left({\hat{\theta }}^{j}-\theta^{j}\right)}^{2}}=\left|{\hat{\theta }}^{j}-\theta^{j}\right|$$ (9)

Table 3 reports the results of model errors before and after calibration. After calibration, position error is included between 0.66 and 15.26 µm while the absolute orientation error is included between 4 $\times 10^{-3}$ mrad and 5.61 mrad. In order to better study the influence of the error made on the values of the intrinsic parameters $\pi_{r}$, the extrinsic parameters $\pi_{ext}$ have been identified even for the nominal model (first step). It shows that the calibration process allows to reduce the position error by a factor of 10 and the orientation error by a factor of 13.

**TABLE 3:** Positioning and orientation errors (respectively $e_{P}$ and $e_{\theta }$) with the nominal or the identified parameters and for the calibration or the validation configuration set.

	$e_{P}$ (µm)			$e_{\theta }\;\left(mrad\right)$		
	Max	Mean	min	Max	Mean	min
Nominal model with calibration set	157.8	70.6	10.1	12.65	4.5	0.058
Identified model with calibration set	15.26	7.19	0.66	5.61	1.7	0.004
Identified model with validation set	13.88	5.5	1.5	5.57	1.5	0.10

### 5.5 Forward Kinematic Model Validation

**FIGURE 9:**
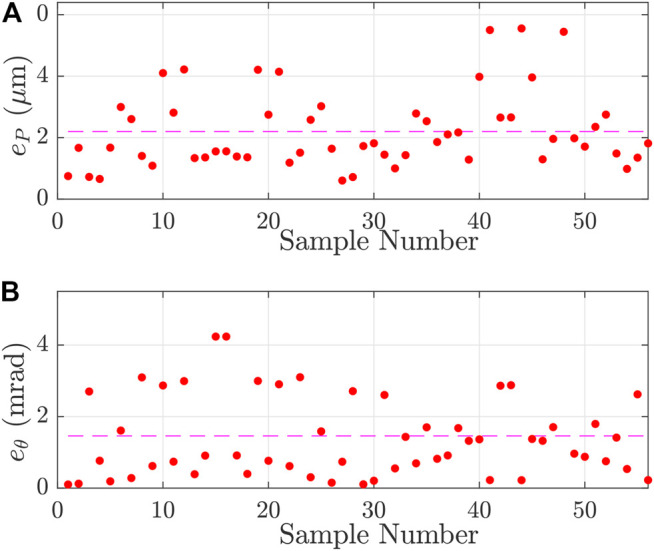
Positioning **(A)** and orientation **(B)** errors of the model (respectively $e_{P}$ and $e_{\theta }$) for the different samples the validation set of joints coordinates. The dashed-lines are the mean values of positioning and orientation accuracy and respectively correspond to 5.5 µm and 1.5 mrad.

To check the validity of the identified forward kinematic model, simulations are performed with a validation set of joints coordinates. Figure 9 shows the obtained results. Position and absolute orientation errors are respectively included between 1.5 and 13.88 µm and between 0.1 and 5.57 mrad. Those results, reported in Table 3, have the same orders of magnitude as those for the calibration set. The small reduction of the position errors is explained by a more compact distribution of poses close to the center of the workspace. The sources of these errors will be discussed in the last section.

For both sets, the maximal resulting position modeling error is 15.26 µm corresponding to 0.05% of the 30 mm length of the flexible rods.

## 6 Robot Accuracy

This section presents the results of the proposed methodology on the PCR’s accuracy. To quantify this accuracy, the robot is controlled in the Cartesian space. This control depends on the inverse kinematic model whose parameters resulted from the calibration process.

### 6.1 Robot Control

**FIGURE 10:**
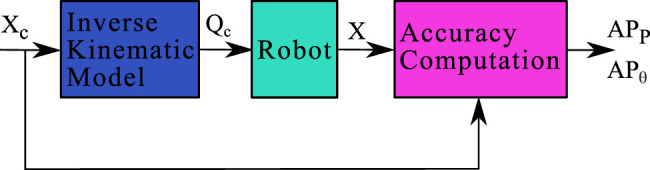
Block diagram of the position control of the robot using the Inverse Kinematic Model.

The forward kinematic model enables the prediction of the pose of the robot considering the joint coordinates as inputs. Conversely, the inverse kinematic model allows to calculate the joint coordinates corresponding to a given pose in the Cartesian space. A regular scheme for controlling the position of the robot in the Cartesian space using the inverse kinematic model is implemented as illustrated in Figure 10. The parameters of the inverse kinematic model are those that have been identified for the forward kinematic model.

To validate the capability of the robot to reach commanded poses in the Cartesian space, two trajectories have been considered: a 2 mm side square and a 3 mm diameter circle with a constant null orientation of the mobile platform. Those trajectories are used as an input for the inverse kinematic model which returns the corresponding joints coordinates $Q_{c}$. Those joints coordinates are sent to the robot and its poses are recorded by the second vision setup (the one composed by a microscope).

**FIGURE 11:**
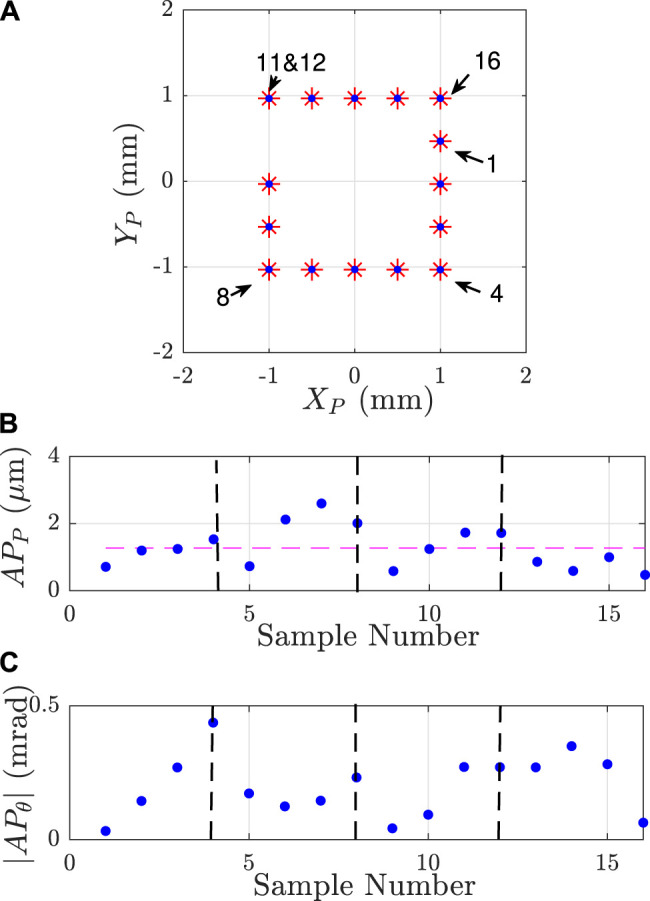
Result of the robot position control for a square trajectory **(A)** commanded poses (in red) and attained by the robot (in blue) **(B)** position and **(C)** orientation accuracies obtained for the different poses. The horizontal dashed-line is the mean value of the positioning accuracy which is 1.27 µm. The black dashed-lines are characteristic points (numbered points) on the trajectories (corners of the square) which correspond to points where the $AP_{P}$ or $AP_{\theta }$ monotony change.

**FIGURE 12:**
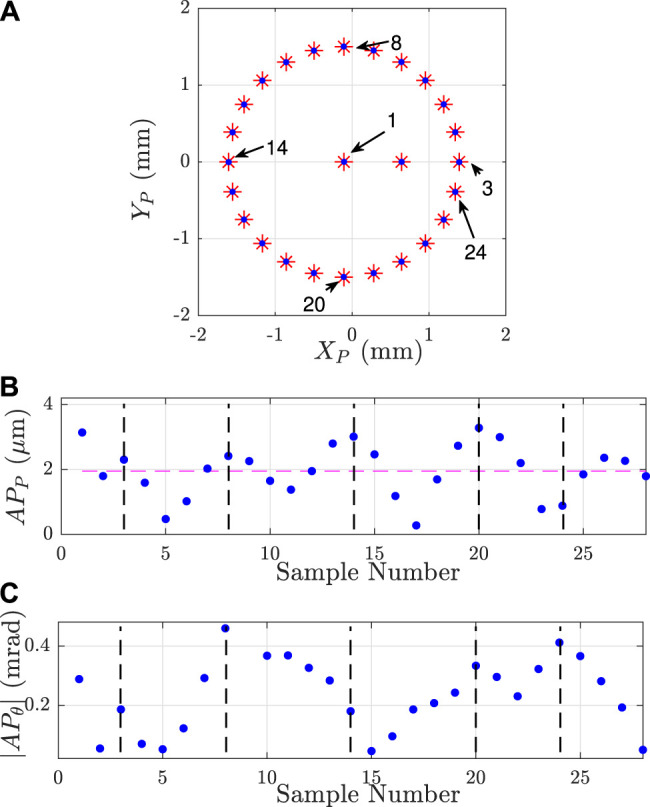
Result of the robot position control for a circular trajectory **(A)** commanded poses (in red) and attained with the prototype (in blue) **(B)** position and **(C)** orientation accuracies obtained for the different poses. The horizontal dashed-line is the mean value of the positioning accuracy which is 1.95 µm. The black dashed-lines are characteristic points (numbered points) on the trajectories where the $AP_{P}$ or $AP_{\theta }$ monotony change.

Figures 11A, 12A show the desired trajectories (red points) and the experimental trajectories (blue points). Both positions and orientations obtained experimentally are closed to the desired ones. The differences between them are too small to be seen at the same time than the performed trajectory. Those results show that the robot is capable of following a defined trajectory.

### 6.2 Evaluation of the Robot Accuracy

The robot accuracy evaluates the closeness of agreement between the pose attained by the robot and its commanded pose. The accuracy of a robot is defined by the standard ISO 9283:1998. The positioning accuracy is the difference between the commanded position and the barycenter of the reached positions:

$$AP_{P}=\sqrt{{\left(\bar{x}-x_{c}\right)}^{2}+{\left(\bar{y}-y_{c}\right)}^{2}}$$ (10)

The orientation accuracy is the difference between the commanded angle and the average of the reached orientations:

$$AP_{\theta }=\left(\bar{\theta }-\theta_{c}\right)$$ (11)

The bar operator ($\bar{\cdot }$) is the barycenter (or the average) of the reached positions (or orientations) after repeating the same pose at least 30 times.

The accuracy of the robot has been evaluated for the two trajectories as shown in Figures 11 (B–C), 12 (B–C). For both cases, the means of positioning accuracy are below 2 µm (1.27 and 1.95 µm) and the worst positioning accuracies are respectively 2.60 and 3.28 µm for the square and circular trajectories. In orientation, the worst orientation accuracy is inferior to 0.532 mrad. Table 4 reports all those results. The resulting mean positioning accuracy is 10.9$\times 10^{-3}$% of the nominal length of a flexible continuum rod (around 30 mm). Considering the performances of the other PCR gathered in Table 1, the presented positioning accuracy is 10 times better than the current relative positioning accuracy.

**TABLE 4:** Positioning and orientation accuracies (respectively $AP_{P}$ and $AP_{\theta }$) of the robot for two different trajectories.

	$AP_{P}$ (µm)			$\left|AP_{\theta }\right|$ (mrad)		
	Max	Mean	min	Max	Mean	Min
Square	2.60	1.27	0.47	0.437	0.20	0.032
Circle	3.28	1.95	0.28	0.532	0.24	0.046

### 6.3 Discussion

For both trajectories (square and circle), the pose accuracies depend on the pose of the platform. For instance, in the square trajectory, the orientation accuracy is worst in the corners. A deeper analysis on the experimental data of the calibration process shows that the angular errors are linked, with a correlation ratio of nearly one, to the differences between the initial angle and the current angle. Larger the orientation of the platform, higher is the angular error. Correlations between the position and the angular error are less straightforward to establish with typical ratios between 0.52 and 0.74. With local studies on different areas of the workspace, there are some locations near the workspace borders were the position errors are increased. In conclusion, the model is more accurate in the middle of its workspace in both orientation and position.

## 7 Conclusion

In this article, a methodology to reach micrometer positioning accuracy is proposed.

This micrometer positioning accuracy was reached thanks to the proposed methodology which consists in five key elements. The first one is the use of a repeatable PCR structure to be able to predict the robot’s quasi-static behavior. The second element is to use a correct mechanical model. The third one is the use of fiducial markers in the design of the robot. Those markers allow to efficiently measure the nominal values of the model’s parameters and to estimate their uncertainties. The fourth element is to conduct a sensitivity analysis to quantify the influence of the different parameters and find the most influential ones. This crucial information is considered in the robot design to be able to efficiently measure those parameters thanks to fiducial markers. The last step is to calibrate the whole robot by identifying all the parameters through three optimization steps. Using this approach on a *XY*Θ planar Parallel Continuum Robot (PCR), the maximal reached positioning accuracy is 3.3 µm in position and 0.5 mrad in orientation over a 10 mm-long circular trajectory.

In addition to the already recognized advantages of PCR (miniaturization capabilities, lightweight, etc.), the obtained results make also PCR worth to consider for high precision positioning tasks.

## Data Availability

The original contributions presented in the study are included in the article/supplementary material, further inquiries can be directed to the corresponding author.
